# Oxygen Uptake Measurements and Rate of Perceived Exertion during a Marathon

**DOI:** 10.3390/ijerph19095760

**Published:** 2022-05-09

**Authors:** Véronique Billat, Luc Poinsard, Florent Palacin, Jean Renaud Pycke, Michael Maron

**Affiliations:** 1 Department of STAPS, Université Paris-Saclay, Univ Evry, 91000 Evry-Courcouronnes, France; veroniquelouisebillat@gmail.com; 2 Laboratory of Neurophysiology and Movement Biomechanics, Université Libre de Bruxelles Neuroscience Institut, 1070 Bruxelles, Belgium; luc.poinsard@gmail.com; 3 UMR8071-CNRS-Laboratoire de Mathématiques et Modélisation d’Evry, Université Paris-Saclay, Univ Evry, 91000 Evry-Courcouronnes, France; jeanrenaud.pycke@univ-evry.fr; 4 Department of Integrative Medical Sciences, Northeast Ohio Medical University, Rootstown, OH 44272 , USA; mbm@neomed.edu

**Keywords:** hitting the wall, self-pace, positive split, cardiovascular drift, physiology of exercise, endurance running

## Abstract

Although the marathon race has been democratized, it remains complex due to the famous “hitting the wall” phenomenon after the 25th km. To characterize this “wall” from a physiological and Rate of Perceived Exertion (RPE) perspective in recreational marathon runners, we report first continuous breath-by-breath gas exchange measurements during an actual marathon race. In order to test the hypothesis that RPE could be a candidate for controlling the marathon pace, this study examined the relationship between RPE and the physiological variables time course throughout a marathon. Only the respiratory frequency and heart rate increased progressively during the race in all the runners, while the oxygen uptake and ventilatory rate followed different kinetics according the individuals. However, the indexation of the physiological parameters and speed by RPE showed the same decreased tendency for all the runners. In conclusion, these results suggest that running a marathon must be self-paced with the RPE.

## 1. Introduction

Participation in marathon running has grown by 100% over the past 20 years [1], with numerous recreational runners taking up the sport. However, after the 25th km, the phenomenon of “hitting the wall” is a familiar experience for 40% of runners throughout marathon history [2]. “Hitting the wall” not only refers to a sudden and dramatic slowing of pace in the latter stages of the race but also to a profound feeling of exhaustion [3]. This phenomenon was previously evaluated in a large-scale data analysis of late-race pacing collapse in the marathon [4]. Smyth presented an analysis of 1.7 million recreational runners, focusing on pacing at the start and end of the marathon, two particularly important race stages.

They showed how starting or finishing too quickly could result in poorer finish times, because fast starts tend to be very fast, leading to endurance problems later, while fast finishes suggest overly cautious pacing earlier in the race [5]. Another study performed with 280 (2 h 30–3 h 40) marathoners, showed that the “fallers” (runners who had a significant decrease in their running speed during the race that appeared at the 26th km) represented the large majority of runners (77%) with significantly lower performance and higher cardiac drift (i.e., an increase of the heart rate/speed ratio) compared with the non-fallers [6]. In addition, marathon performance was correlated with the amplitude of this cardiac drift, which was very sensitive to the running strategy.

The analysis of this running strategy showed that the faller group ran more than half the marathon distance (56%) above their average speed before they “hit the wall”. Hence, the choice of pace remains a delicate issue for the recreational marathoners, and research must explore further regarding our comprehension of the physiological responses during a marathon [6]. This race of 42,195 km elicits a high percentage of cardiac output and $\dot{V}$O2 max for more than 2 h 30 min [7,8,9,10]. This capacity of sustaining a high fraction of maximal cardiac output and $\dot{V}$O2max (qualified of “Endurance”) has been reported to be correlated with the marathon performance [6,10,11,12,13,14].

However, to date, only one experiment, 46 years ago, measured the gas exchange and respiratory rate during the race [8], and only one study reported the Rate of Perception of Exertion (RPE) during a marathon [15]. The Borg 6–20 RPE Scale [16] is a reliable measure used to quantify, monitor and assess the capacity and levels of exertion due to strong correlations between the RPE and physiological parameters [17].

A study [15] showed that the RPE time course was not modified by the ingestion of carbohydrates compared with placebo beverages; however, the runners were able to run faster with the same RPE throughout the race. These results suggest that RPE could be a candidate for controlling marathon pacing; however, the question remains regarding the trends and breakpoints of the RPE with each cardiorespiratory variable. Hence, the main purpose of this study was to test the hypothesis that (1) sudden changes in the physiological variables and RPE occurred when the recreational marathoners “hit the wall” and (2) that early drifts of the cardiorespiratory (HR, $\dot{V}$O2, …)/RPE ratio could appear early in the race.

## 2. Materials and Methods

### 2.1. Subjects

Our subjects were nine male, non-elite marathon runners (mean ± standard deviation (SD) age: 40.1 ± 10.6 years; weight: 72.7 ± 6.5 kg; and height: 178.3 ± 7.5 cm) (Table 1). For organizational reasons, we chose to have only one gender in the present study in order not to introduce an additional factor that could influence the statistical analysis. All the subjects volunteered to participate in the study, and we asked them to not modify their habitual training.

All runners had previously run at least two marathons and had regularly trained three to four times per week (50–80 km/week) for more than 5 years. Once a week, they performed a High Intensity Interval Training of 6 × 1000 m at 90–100% of their maximal heart rate and a tempo training (15–25 km) at 100–90% of their average marathon speed. The study’s objectives and procedures were approved by an institutional review board (CPP Sud-Est V, Grenoble, France; reference: 2018-A01496-49). All participants were provided with study information and gave their written consent to participate.

### 2.2. The Marathon Race

All participants ran a marathon in an official race (Sénart Marathon, France). The start was at 9 am, and for the environmental conditions on 1 May 2019 in Sénart, the temperature was between 11 and 15 °C (between 9 a.m. and 1 p.m.), there was no precipitation, and the humidity in the air averaged 60%. The blood lactate was measured on the finger (Lactate PRO2 LT-1730; ArKray, Japan) right after the runners’ warm up (15 min at easy pace) and at the third minute after they crossed the finish line.

### 2.3. Experimental Measurements

Respiratory gases (oxygen uptake [$\dot{V}$O2], ventilation [$\dot{V}$E] and the respiratory exchange ratio [RER]) were continuously measured using a telemetric, portable, breath-by-breath sampling system (K5; Cosmed, Rome, Italy). A global positioning system watch (Garmin, Olathe, KS, USA) paired with the K5 system was used to measure the HR and the speed response (using 5 s data averages) throughout each trial. We used the same cardiac belt for the Garmin and K5 because it was compatible for both. Given that it has been recently shown that marathon performance depends on pacing oscillations [18], we encouraged the runners to self-pace their run without focusing on the cardio-GPS whose dial was hidden.

During the marathon, refreshment points (offering water, dry and fresh fruit and sugar) were located every 5 km as well as at the finish line, and sponge stations were every 5 km from km 7.5. At the aid stations, the runners were allowed to remove their masks so they could drink or eat. All the runners drank one glass at each hydration point (with flat water and fruits) positioned on every 5 km of the route and in the Start/Finish area.

COSMED reusable face masks are ideal for metabolic testing both at rest and during exercise, regardless of the nature, intensity or duration of the test. These masks are made of silicone (without latex or other allergenic materials) and are anatomically contoured with a strong ribbed support structure and an integrated chin strap to ensure a perfect fit with no leakage and to provide maximum comfort even if it does not have any impact on the data accuracy. To improve the comfort, the runners used the mask version with inspiratory valves that reduce inspiratory resistance during high-intensity exercise.

### 2.4. The Rate of Perception of Exertion

The RPE was recorded by the runners using a small microphone that they carried with them. The runners recorded an RPE at least every km or more frequently if they felt the need. We used the Borg 6–20 scale [16] scale to assess fatigue during the marathon as a correlate to the physiological stress indicators. The runners were familiarized with the scale during the 2 weeks preceding the race.

### 2.5. Statistical Analysis

All statistical analyses were performed using XLSTAT software (version 2019.1.1, Addinsoft, Paris, France). All the test variables ($\dot{V}$O2, $\dot{V}$CO2, HR and speed) were reported as the mean ± SD. For each variable, the normality and homogeneity of the data distribution were examined using a Shapiro–Wilk test.

#### 2.5.1. Global Tendency of Pace and Its Asymmetry

The trend in speed time series (i.e., Kendall’s τ non-parametric rank correlation coefficient) [19] and the pacing design (i.e., asymmetry characteristics of the race) [20] were compared. The equation of Kendall’s τ:

(1) $$\tau =\frac{2}{n(n-1)}\sum_{i<j}K\left(v_{i},v_{j}\right)$$

$v_{i}$ = *i*th value of a speed; $v_{j}$ = *j*th value of a speed; *i* < *j* = *i* indicates a period of time prior to *j*; sum being performed over the *n*(*n*− 1)/2 distinct unordered couples of indices i,j, so that τ takes values in between −1 and 1.

Furthermore, we calculated the skewness value of the speed distribution as 3 (mean – median)/SD. The skewness is a measure of the asymmetry of the probability distribution of a real-valued random variable about its mean. A negative coefficient indicates a distribution shifted to the right of the median and thus a distribution tail spread to the left.

Its value can be positive, negative or undefined: a positive skew means that the mean is greater than the median, while a negative skew means the mean is less than the median. In that case, it means that the marathoner run more kilometres above the final average speed because of the high speed decrease in the final part of the race [20,21]. More specifically, we compared the time spent below the average speed with the time spent above the average speed. Positivity asymmetry was defined as a run with 54% or more of the time spent below the average speed, and negative asymmetry was defined as a run with 46% or less of the time spent above the average speed.

#### 2.5.2. Tested “Ascending” and “Descending” Series

This test makes it possible to detect a gradual deviation (as the sample is studied) from the mean of the distribution considered not only when it has a monotonic character but also in the more general case, for example, of a periodic trend. The sequence of plus and minus signs is studied. Precisely, we start from the set of observation results, from the sample x1, x2, …, xn. At the i-th rank, we place the plus sign if xi + i − xi > 0, and the minus sign if xi + i − xi > 0 (if two or more observation results are equal, only one of them is taken into account).

It is clear that a sequence of plus signs will correspond to the growth of the observation results (“ascending series”) and a sequence of minus signs to the decrease (“descending series”). If the sample is random (the observations are independent), the global number of series in the sequence of signs that we formed cannot be very small, just as the length of these series (the number of signs of the same nature) cannot be too large. In particular, for the significance level 0.05 < α < 0.10.

(2) $$\left\{\begin{array}{c} v\left(n\right)>\frac{1}{3}\left(2n-1\right)-1,96\sqrt{\frac{16n-29}{90}}\\ \tau \left(n\right)<\tau_{0}\left(n\right) \end{array}\right.$$

$\tau_{0}\left(n\right)$ is defined as :

(3) $$\underset{\tau_{0}\left(n\right)}{n}\;\underset{5}{n\;⩽\;26}\;\underset{6}{26\;<\;n\;⩽\;153}\;\underset{7}{153\;<\;n\;⩽\;1170}$$

If at least one of the inequalities (2) is not satisfied, the hypothesis that the sample was randomly selected must be rejected. Considering the number of data of 84 (one analysed this series averaging the data every 500 m, hence 2×42 = 84 data), hence *n* < 153, then we consider the value of τ = 6 the minimum required for considering that beyond the global tendency of the time series identified by Kendall’s τ, there is a systematic increase or decrease at this local 500 m of the marathon. This aimed for identifying the famous “hitting the wall” applying this systematic test on all the cardiorespiratory and speed characteristics (cadence). In addition, we compared each 5 km applying a Student’s t test for paired data.

#### 2.5.3. Multivariate Data Analysis

We detected possible multicollinearity between the variable of interest: HR, Rf, speed, cadence and RPE. Before the multivariate analysis, we calculated the variance inflation factor (VIF) measuring the amount of multicollinearity in a set of multiple regression variables.

(4) $$VIF=\frac{1}{1-R_{i}^{2}}$$

Mathematically, the VIF for a regression model variable is equal to the ratio of the overall model variance to the variance of a model that includes only that single independent variable. This ratio is calculated for each independent variable. Statistical software (XLSTAT) calculates a VIF for each independent variable. VIFs start at 1 and have no upper limit. A value of 1 indicates that there is no correlation between this independent variable and any others. VIFs between 1 and 5 suggest that there is a moderate correlation; however, it is not severe enough to warrant corrective measures.

VIFs greater than 5 represent critical levels of multicollinearity where the coefficients are poorly estimated, and the *p*-values are questionable [22]. That is why we retained the variables who had a value of VIF > 5 to perform a multivariate analysis with a Principal Component Analysis. For more transparencies, given that we wanted to applied this multivariate analysis using the same variables, which showed a real tendency in the time series for all the runners (Rf, speed, Tidal volume RPE, HR, cadence) indicated their VIF.

#### 2.5.4. Principal Component Analysis

Principal Component Analysis is one of the most frequently used multivariate data analysis methods for dimensionality reduction. It is a projection method as it projects observations from a p-dimensional space with p variables to a k-dimensional space (where k < *p*) so as to conserve the maximum amount of information (information is measured here through the total variance of the dataset) from the initial dimensions. PCA dimensions are also called axes or Factors. If the information associated with the first two or three axes represents a sufficient percentage of the total variability of the scatter plot, the observations could be represented on a 2 or 3-dimensional chart, thus making interpretation much easier. PCA is considered to be a Data Mining method as it allows to easily extract information from large datasets.

There are several uses for it, including: the study and visualization of the correlations between variables to hopefully be able to limit the number of variables to be measured afterwards. We obtained non-correlated factors, which are linear combinations of the initial variables, to use these factors in modelling methods, such as linear regression, logistic regression or discriminant analysis. Visualizing observations in a two or three dimensional space in order to identify uniform or atypical groups of observations.

We chose the Pearson, (the classic PCA, that automatically standardizes or normalizes the data prior to computations to avoid inflating the impact of variables with high variances on the result). PCA is one of the most frequently used multivariate data analysis methods for dimensionality reduction. We then examined the variability captured on two orthogonal vectors (F1 and F2). The correlation circle (or variables chart) shows the correlations between the components and the initial variables. Principal Component Analysis is one of the most frequently used multivariate data analysis methods for dimensionality reduction. We examined the variability captured on two orthogonal vectors (F1 and F2).

The correlation circle (or variables chart) shows the correlations between the components and the initial variables. The first map is called the correlation circle (below on axes F1 and F2). It shows a projection of the initial variables in the factors space. When two variables are far from the centre, then, if they are: Close to each other, they are significantly positively correlated (r close to 1); If they are orthogonal, they are not correlated (r close to 0); If they are on the opposite side of the centre, then they are significantly negatively correlated (r close to −1). Vectors are the loadings on PC1 (x-axis) and PC2 (y-axis).

Vector length indicates the strength of the relationship and the angle between two vectors gives the degree of correlation (adjacent = highly correlated, orthogonal (90°) = uncorrelated and opposite (180°) = negatively correlated). Agglomerative Hierarchical Clustering (AHC) was then applied, and the agglomerative hierarchical-clustering dendrogram shows the progressive grouping of the individual physiological responses during the marathon. The process starts by calculating the dissimilarity between the N objects.

Then, two objects, which when clustered together minimize a given agglomeration criterion, are clustered together thus creating a class comprising these two objects. Then, the dissimilarity between this class and the N-2 other objects is calculated using the agglomeration criterion. The two objects or classes of objects whose clustering together minimizes the agglomeration criterion are then clustered together. This process continues until all the objects have been clustered. These successive clustering operations produce a binary clustering tree (dendrogram), whose root is the class that contains all the observations.

This dendrogram represents a hierarchy of partitions. It is then possible to choose a partition by truncating the tree at a given level, the level depending upon either user-defined constraints (the user knows how many classes are to be obtained) or more objective criteria. The full dendrogram displays the progressive clustering of objects. If truncation has been requested, a broken line marks the level the truncation has been carried out. The truncated dendrogram shows the classes after truncation.

## 3. Results

Each marathoner finished the race, and three of them ran their personal best times despite carrying the weight of the devices (Table 1).

The final blood lactate value was equal to 2.8 ± 0.7 vs. 1.8 ± 0.8 mM after warm up phase (*p* < 0.05). All the marathoners had their final blood lactate value set between 2.1 ± 3.8 mM.

### 3.1. Trend and Asymmetry Characteristics of Speed and Cardiorespiratory Variables in the Marathon Race

- Speed:
  All but one (runner 2), marathon runners ran a large positive split race as indicated by a negative Kendall’s τ (Table 2). The speed highly decreased throughout the race considering this global tendency. Furthermore, they had a positive skewness and a negative asymmetry (Table 2). A negative coefficient indicates a distribution shifted to the right of the median and thus a distribution tail spread to the left. More specifically, the speed factors: cadence and amplitude, also followed the same significantly negative trend (Table 2). All these runners (but the runner 2) ran more of the average distance above average speed (56 ± 3%).

- Cardiorespiratory time series tendencies:
  There were different tendencies between the cardiorespiratory parameters as well as in the runners. In all runners (except runner 6), the respiratory rate showed a highly significant positive trend, while the tidal volume decreased. The minute ventilation increased (3/9) or decreased (6/9) depending on the balance between the respiratory rate increase and tidal volume decrease.
  Similarly, $\dot{V}$O2 decreased in two thirds of the marathoners (6/9) but $\dot{V}$CO2 and RER decreased in almost all (8/9) of the runners. The physiological variables indexed by the speed gave a clearer picture than the physiological variables alone, due to the decrease in speed. For example, Rf/speed decreased in every runners (9/9), including one who had a decrease in Rf (R4). However, expressing the variables as a function of the speed did not change the patterns observed for the other variables (HR, $\dot{V}$O2, $\dot{V}$CO2, Vt and VE) (Table 3). When we expressed the variables as a function of the RPE, we saw that all the variables showed a negative trend in all the runners (Table 4). Now, after having identified this global tendency, we attempt to identify some point distance where the variables change more abruptly.

### 3.2. Tested “Ascending” and “Descending” Series

This “ascending” and “descending” series test reveals that no marathoners showed an abrupt decrease of their speed. Runner 3, however, exhibited a significant decrease in speed very early in the race (at the 7.5th km).

In contrast, the cadence decreased abruptly in more than half of the runners (5/9) after the 25th km, and in half of them, this cadence drop was already apparent before the tenth km. The cardiorespiratory variables did not have any abrupt decrease during the race when we attempted to identify this by examining each 10 s average value by the ascending and descending methods. The cadence’s drop was not associated with those of the cardiorespiratory variables.

### 3.3. Comparison of Each 5-km Splits (t-Student Test for Paired Data)

**Figure 1:**
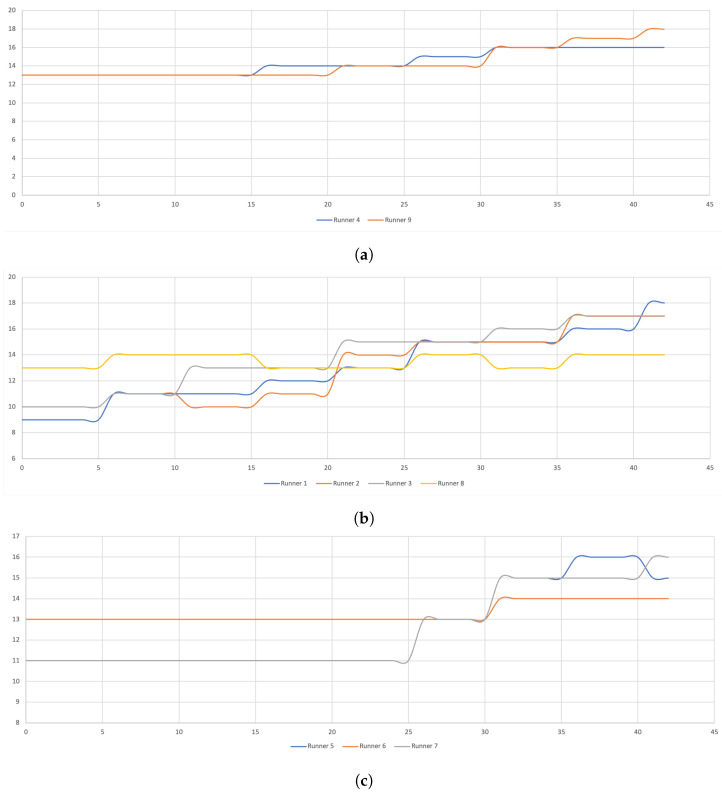
Rating of Perceived Exertion during the marathon. In the (**a**) group of runners the RPE increased once at the fifth km, in the (**b**) group runners RPE increased at the 15–20th km and (**c**) group runners RPE increased only at the 30th km. Among this last group, we notice runner 6 who had only one RPE increase for the entire marathon.

RPE increased every 10 km while, the speed decreased every 5 km from the fifth km until the end of the race (Table 5). This evolution did not follow the same dynamics in each individual, even if we could distinguish three groups of runners with almost the same tendencies (Figure 1). The first group of runner increased RPE after the fifth km, while the others two did this at the 15–20th km and at the 30th km, respectively (Figure 1). Among this last group, it can be noticed the runner 6 had only one RPE increase during the marathon race.

- Cardiorespiratory responses: heart rate increased at the fifth km and then, stabilised until the 15th km where it increased again and then stabilise again until the end the marathon. $\dot{V}$O2 remained stable in contrast with $\dot{V}$CO2, which decreased at the tenth and the 30th km resulting in a decreases in the respiratory exchange ratio (RER).

### 3.4. Multivariate Analysis for Cardiorespiratory and Speed Variables

**Figure 2:**
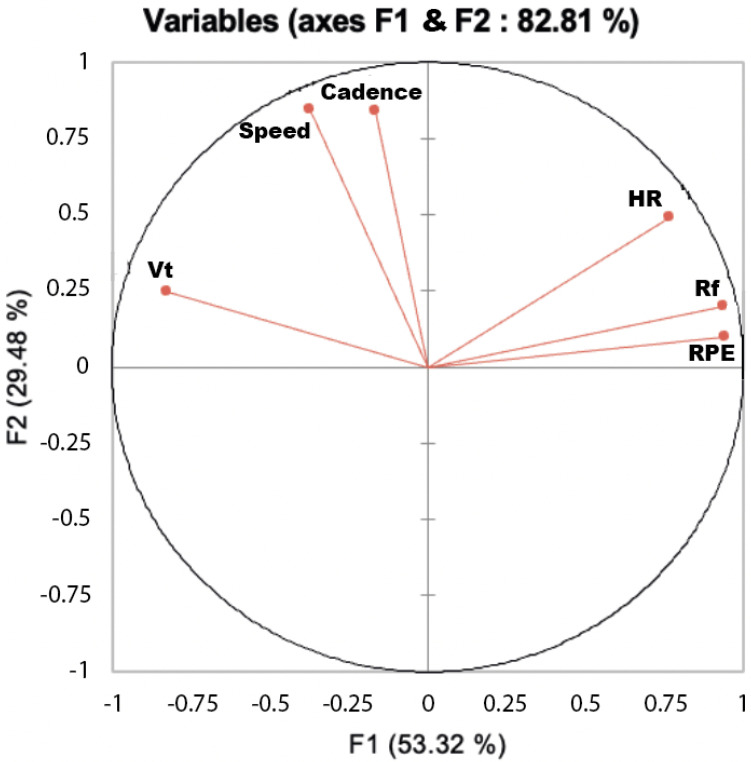
Correlation circle for unrotated principal component analyses (PCA) on each physiological and cadence responses during the marathon for the runner 3 who is representative of all the nine marathoners.

The first correlation circle shows that, for the runner number 3 (as for all the runners) RPE and Rf are closed (their angle is acute) (Figure 2). The horizontal axis F1 is rather linked with RPE and Rf, and the vertical axis F2 is the linked with speed, heart rate and Tidal volume (Figure 2).

**Figure 3:**
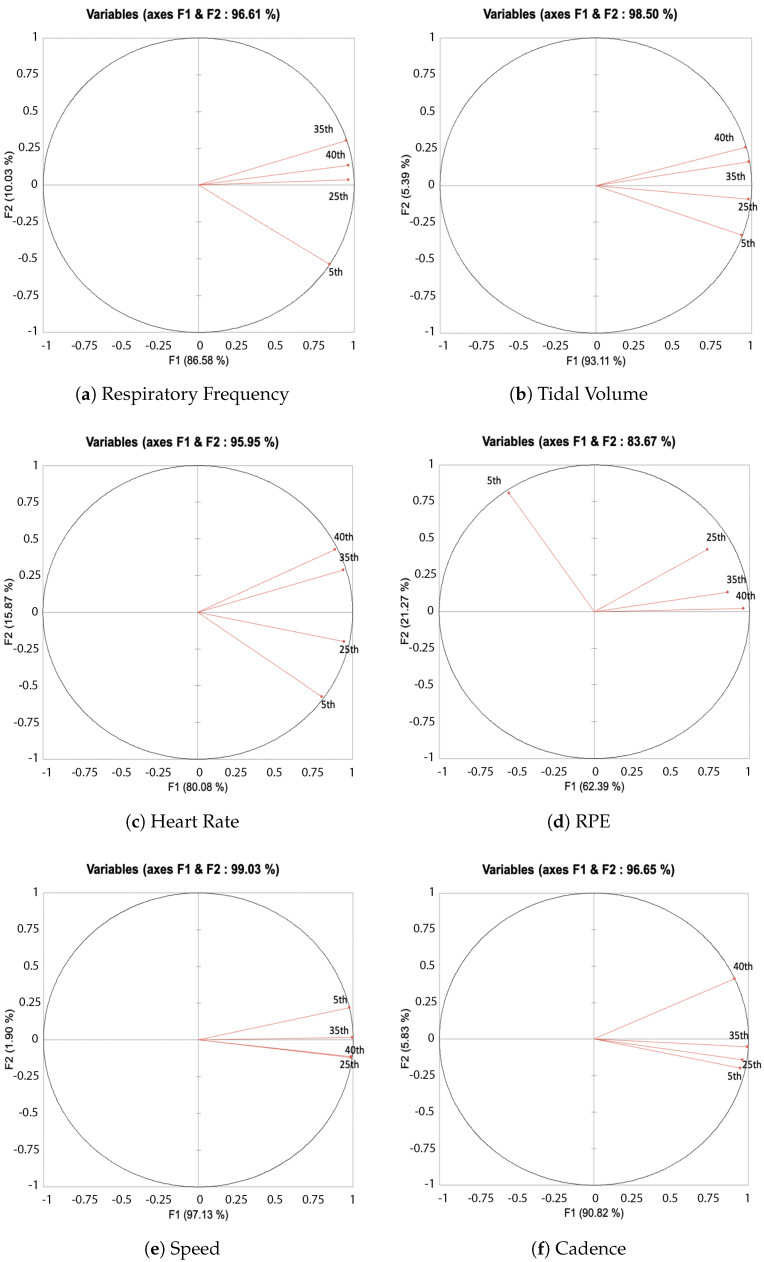
Correlation circles for unrotated principal component analyses (PCA) on each physiological and cadence responses during the 5 km part of the marathon. Vectors are the loadings on PC1 (x-axis) and PC2 (y-axis).

The multivariate analysis for 5-km splits shows that for Rf and RPE, the first 5 km is clearly different from the 25th, 35th, 40th km (Table 6). This was not the case for the others variables: cadence, speed, heart rate and the tidal volume (Figure 3).

**Figure 4:**
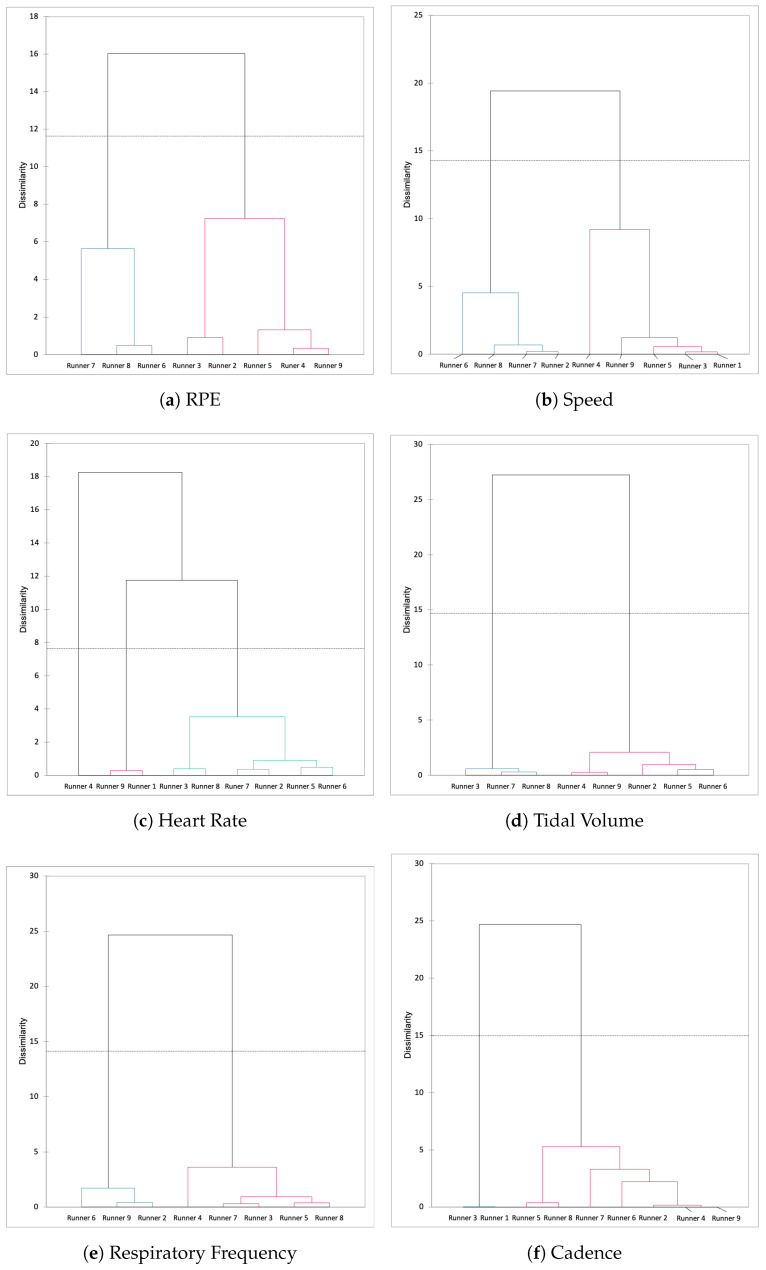
Dendogram for the classification of different runners’ profile according their physiological response during the marathon.

When we analyse the correlation circle for each variables When we classified the runners according to these variables each 5 km, we could see that the classification was the same for RPE and speed and that runners 6, 7 and 8 formed a cluster (Figure 4). Other variables did not allow us to identify a cluster of runners.

## 4. Discussion

**Figure A1:**
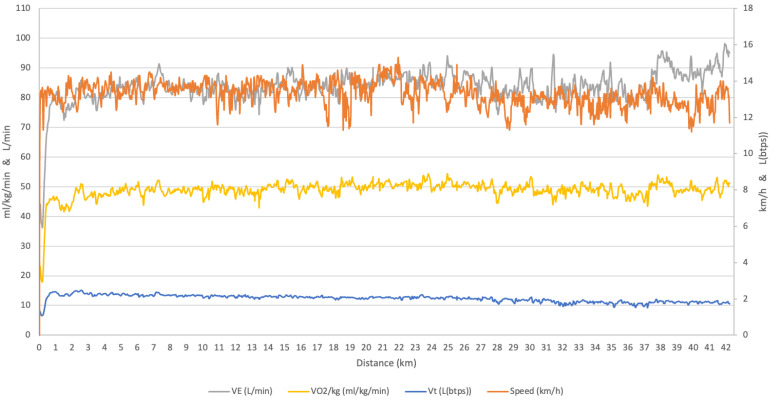
$\dot{V}$E, $\dot{V}$O2, Vt and speed during the marathon.

To the best of our knowledge, this is the first study to report the continuous measurement of cardiorespiratory function during a marathon race. Although the marathon race has been democratized, it remains complex due to the famous “hitting the wall” phenomenon after the 25th km. To characterize this “wall” from a physiological and Rate of Perceived Exertion (RPE) perspective in recreational marathon runners, we report the first continuous breath-by-breath gas exchange measurements during an actual marathon race (Figure A1).

**Figure A2:**
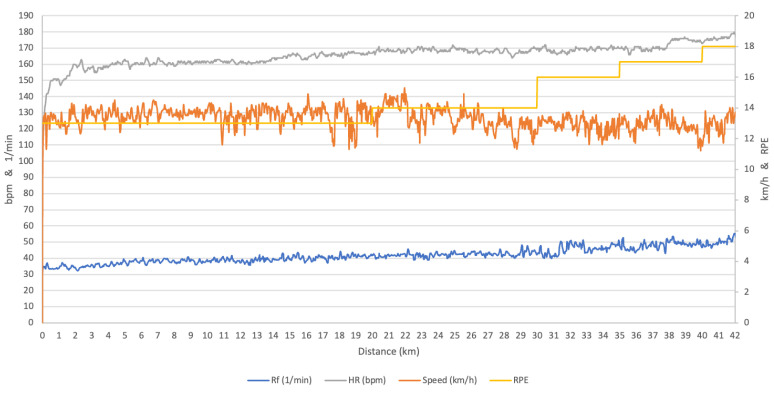
Rf, HR, RPE and speed during the marathon.

During the marathon, the oxygen uptake and ventilatory rate presented different kinetics according the runners, The sole sudden change in the physiological variables occurred when the recreational marathoners “hits the wall”, was those of the respiratory rate. However, the indexation of the cardiorespiratory parameters by RPE ratio showed a systematic drift throughout the race. The multivariate analysis of cardiorespiratory variables matrix revealed that RPE was projected on the same axis than the respiratory frequency suggesting that RPE is directly connected with this cardiorespiratory variables (Figure A2).

In addition, the systematic increase of the respiratory frequency in all but one runner led us to hypothesize that these are good candidates for pace regulation during a marathon [17]. The marathoners ran a RPE = 13.9, a value below their anaerobic threshold. The final blood lactate accumulation during the marathon was in accordance with those already reported by Costill in their seminal paper about the physiology of the marathon [10].

Indeed, he underlined the marathoner’s ability to maintain an extremely high rate of energy expenditure for 2.1 to 3.0 h is impressively demonstrated by one runner, i.e., Derek Clayton, who ran in 2 h 8 min 33 s with a final lactate value at the relatively low level of 21.4 mg/100 mL (2.4 mM/100 mL).

The analysis of speed time depended on the GPS but showed a large speed variation [6,20], and the marathon performance depended on pacing oscillations between nonsymmetric extreme values [18]. Beyond this analysis of the pace and HR time series during a marathon, the present study aimed to highlight the possible relationship between this pacing variation with those of the cardiorespiratory variables and with RPE. We found that, after the 25th km, “hitting the wall”, was expected to appear.

Big data on much more subjects are necessary for distinguish different marathon pace strategy taking into account only the time split every 5 km (2295 and 31,762 runners [23,24]). Our small sample of marathoners had homogeneous pace time serie but with very different physiological and RPE responses. Their pacing were homogeneous and consistent with that reported in the literature for the recreational marathon runners category [5,21,25,26].

It has been shown pacing strategies, in addition to the physiological aspect, include psychological aspects, such as coping mechanisms, self-esteem and life meaning [25]. That is why the runners crossed the RPE and the physiological and pacing (speed) time series during the marathon. Indeed, the perception of effort is multidimensional and is governed by many psychological and experiential factors [27].

Various terms and phrases, such as perceived exertion, perception of effort, sensation of effort, exertion effort and effort sense have been employed synonymously by investigators in this area of inquiry [27]. Currently, RPE, which is now largely familiar to coaches and increasingly by endurance athletes and it appears that, for a self-paced test based on RPE, a race, such as the marathon, can also be controlled by RPE recommendations.

Individually, the performance template, as the pattern of power output during self-pace exercise has been shown to be robust and regulated in an anticipatory manner [28]. Then, the individual performance template could also be or not be associated with a physiological and RPE [17,29]. The influence of time and distance left to complete, which depends on the speed at each instant, will influence the pacing according physiological variables [17,29]. That is why we focused on the time series of the ratio between physiological variables, candidates for the role of negative feedback controllers—the so-called “homeostats” [30].

The coefficient of variation for the running speed was consistent with that observed in of a prior study performed on 280 marathon runs [20]. In the present study, all the marathoners (but one) ran in “positive split,” that is to say, with a speed decrease trend in accordance with prior study with an extremely low value of risk α (*p* < 0.0001) associated with the same tendency for the both components of the runners’ speed. It was recently shown that the variation in running velocity did not affect the aerobic cost of running at speed below anaerobic threshold [31].

More importantly, our marathoners did not optimize their races in regard to speed asymmetry as they ran more than 56% of the distance above their average speed. This had consequence on the cardiorespiratory variables according their time series directional trend but not in the same way for all runners. Indeed, while heart rate has a positive or negative trend among runners, in contrast with prior studies reporting a systematic cardiac drift during a marathon [7], the respiratory rate had a highly significant positive trend for all runners but not the minute ventilation due the tidal volume decreasing in all the runners.

In a previous study, that measured the cardiac output throughout a marathon, they did not observe this compensation phenomenon between frequency (HR) and volume (SV). Indeed, the cardiac output remained at 83 ± 20% of the maximal cardiac output with a stroke volume at 77 ± 11 and an HR at 87 ± 6% a $\dot{V}$O2 at 76 ± 8% of and a cardiac output at 83 ± 20 of their maximal values [6]. In contrast to the decreasing tidal volume observed in this study, in our previous study, the stroke volume did not decrease over time, which agrees with previous reports of steady state in SV during large reductions in brain perfusion in the heat-stressed human and during intense, endurance exercise [32]. Indeed, SV remained at submaximal steady state (77 ± 3%), as did CO (69 ± 3%).

Furthermore, an increase in HR in a neutral environment has been shown to be responsible for the SV decline in steady exercise performed for 1 h at 57% of $\dot{V}$O2max [33]. As observed in our previous study, of the time course of the cardiac-output during a marathon, the cardiac cost, i.e., (beat/m) increased in conjunction with the increase in HR and decrease in speed. In this study, we did not measure the cardiac output as in this prior study (Billat et al., 2012 [7]), but we chose to measure the cardiorespiratory exchanges. Indeed, cardiac output (physioflow, Manatec, France) device required many probes, and the runners were already encumbered with a cardiac belt and the 1 kg of device to measure $\dot{V}$O2.

In addition, it is important to emphasize that the most important factor is the quality of the data, which is a real challenge such natural and long-term exercise endeavors as climbing the Mont Blanc (Billat et al., 2010 [34]). Indeed, the problem of the long and intense run as the marathon is to have good data despite the sweat that loosens the sensors and makes the heart rate belt slip.

The absence of an abrupt decrease of the runners speed showed that they did not hit the wall if we only consider this face of the definition. While we detected no abrupt speed decrease, we observed a decrease in cadence in more than half of the runners (5/9) after the 25th km. Therefore, the consideration of only speed and the stride parameters analysis, especially at constant speed, does not allow us to detection of abrupt changes. The cardiorespiratory variables also did not, show an abrupt change according to the ascending and descending statistical series method. Again, we observed highly individual cardiorespiratory and speed or cadence responses at very different distances during the race.

This is why we attempted to detect differences by considering the race splits as is more commonly done, every 5 km of the race [35] but without selecting a steady state phase since our objective was to search for steady state breaks that could allow us to detect the famous marathon wall.

The multivariate analysis revealed that, for all the runners, RPE and Rf are closed (their angle is acute) while the speed is closer to the cadence and to the Tidal volume. There are two distinguished axes, one (horizontal) rather linked with RPE and Rf and the other (vertical) with the speed, heart rate and Tidal volume. Then, RPE was closed to Rf while speed, cadence and Hr were aggregated on the orthogonal to F1 (F2) axis.

This type of multivariate analysis could be interesting to be applied on much more subjects taking these parameters (cadence, HR, Rf, RPE, speed) to test the hypothesised that some runners have a RPE-Rf-sensitive profile, while others are sensitive to the cadence and tidal volume. We added the runners as observations above the layer of physiological and speed variables. For that, when we classified the runners according the variables each 5 km, we could see that the classification was the same for RPE and speed, and the runners 6, 7 and 8 formed a cluster.

We could not distinguish any similitude between the dendograms of the other variables. In the same way, we tested the hypothesis than some 5-km splits are characterised by the same time series trend. Therefore, RPE was closed to Rf while speed, cadence and Hr were aggregated on the orthogonal to F1 (F2) axis. This type of multivariate analysis could be interesting to apply on more subjects with these parameters (cadence, HR, Rf, RPE and speed) integrated in an algorithm to define the specific types of marathon runner.

## 5. Conclusions

Even though the majority of runners simply want to complete a marathon without having to focus too much on their time, they also want to be able to enjoy the experience without suffering and hitting the wall. Beyond the volume of training, which has been shown to be significantly correlated with the marathon finish time [36], the choice of the intensity with respect to the specificity of the marathon must be refined. The first take-home message is that a global average analysis on a group of runners, even if they have the same performance level, hides individual differences in response.

Thus, training must be personalized as much as possible according to each subject’s adaptation during such a long and intensive run. This may be more important in recreational runners than in elite athletes who have optimised their race by their ability to pace oscillations likely due to their high power reserves allowing them to recover and to achieve a higher average speed rather than to run a race at a strictly constant pace [18]. Indeed, the cardiorespiratory factors measured in this study exhibited a variable fractional use of maximal values.

Accordingly, we must address the question of the physiology of marathon running in recreational runners on an individual basis. The RPE could be a candidate for controlling the marathon pace given that the indexation of the physiological parameters or speed by RPE showed the same decrease tendency for all the runners. In conclusion, these results suggest that the running of a marathon must be self-paced with the RPE. It is precisely because of this lack of time that we attempted to provide some new insights about the specificity of physiological training according to pace and RPE to help runners to optimize their training. 

## Figures and Tables

**Table 1:** The subjects age, personal best marathon time and the year of this performance. * Runner who performed their personal best during the Sénart marathon.

n° Runners	Age (Years)	Fastest Marathon Times (Years)	Sénart Marathon (2019)
1	47	03 h 12${}^{\prime }$48${}^{\prime \prime }$ (2016)	03 h 31${}^{\prime }$34${}^{\prime \prime }$
2	44	03 h 34${}^{\prime }$57${}^{\prime \prime }$ (2019)	03 h 34${}^{\prime }$57${}^{\prime \prime }$ *
3	22	03 h 22${}^{\prime }$40${}^{\prime \prime }$ (2019)	03 h 22${}^{\prime }$40${}^{\prime \prime }$ *
4	34	02 h 50${}^{\prime }$00${}^{\prime \prime }$ (2019)	02 h 50${}^{\prime }$00${}^{\prime \prime }$ *
5	47	02 h 59${}^{\prime }$22${}^{\prime \prime }$ (2016)	03 h 32${}^{\prime }$07${}^{\prime \prime }$
6	58	03 h 27${}^{\prime }$32${}^{\prime \prime }$ (2013)	04 h 30${}^{\prime }$34${}^{\prime \prime }$
7	29	02 h 57${}^{\prime }$03${}^{\prime \prime }$ (2015)	03 h 14${}^{\prime }$24${}^{\prime \prime }$
8	36	03 h 27${}^{\prime }$58${}^{\prime \prime }$ (2017)	03 h 51${}^{\prime }$44${}^{\prime \prime }$
9	43	02 h 44${}^{\prime }$00${}^{\prime \prime }$ (2015)	03 h 13${}^{\prime }$53${}^{\prime \prime }$

**Table 2:** Trend and asymmetry (Skewness: Sk) characteristics of speed and cardiorespiratory variables in the marathon race (increasing: ↗, constant: ⟶ or decreasing: ↘). % km (% of kilometers ran below the mean speed race).

n° Runner		*Rf*	*Vt*	$\dot{V}$ *E*	*Cad*	*HR*	*Speed*	$\dot{V}$*O*2	$\dot{V}$*CO*2
Runner 1	Trend	↗	↘	↘	↘	↗	↘	↘	↘
	Kendall Tau	0.42	−0.70	−0.46	−0.24	0.43	−0.54	−0.25	−0.59
	*p*-value	0.001	0.001	0.001	0.001	0.001	0.001	0.001	0.001
	Sk Pearson	−0.43	0.84	−0.29	−0.30	−0.08	0.05	−0.50	0.29
	% km	41	56	78	45	49	36	47	61
Runner 2	Trend	↗	↘	↘	↘	↘	↗	↘	↘
	Kendall Tau	0.63	−0.67	−0.39	−0.28	−0.003	0.07	−0.19	−0.30
	*p*-value	0.001	0.001	0.001	0.001	0.883	0.001	0.001	0.001
	Sk Pearson	0.36	−0.12	−0.18	−0.08	0.05	0.50	0.29	0.05
	% km	56	49	48	49	51	40	50	22
Runner 3	Trend	↗	↘	↗	↘	↗	↘	↗	↘
	Kendall Tau	0.71	−0.62	0.42	−0.35	0.66	−0.34	0.20	−0.04
	*p*-value	0.03	0.001	0.001	0.001	0.001	0.001	0.001	0.03
	Sk Pearson	0.28	−0.37	−0.21	−0.17	−0.30	−0.89	−0.48	0.04
	% km	52	47	46	45	43	38	41	73
Runner 4	Trend	↗	↘	↗	↘	↘	↘	↗	↗
	Kendall Tau	0.40	−0.40	0.15	−0.48	−0.12	−0.45	0.01	0.02
	*p*-value	0.001	0.001	0.001	0.001	0.001	0.001	0.41	0.32
	Sk Pearson	1.09	−0.36	−0.01	−0.23	−0.12	−0.70	−0.24	0.34
	% km	68	45	49	47	48	22	44	53
Runner 5	Trend	↗	↘	↘	↘	↘	↘	↘	↘
	Kendall Tau	0.15	−0.36	−0.14	−0.19	−0.21	−0.57	−0.076	−0.35
	*p*-value	0.001	0.001	0.001	0.001	0.001	0.001	0.001	0.001
	Sk Pearson	−0.93	0.41	−0.32	−0.22	0.08	0.01	−0.44	−0.11
	% km	29	56	45	45	51	61	42	35
Runner 6	Trend	↘	↘	↘	↘	↗	↘	↘	↘
	Kendall Tau	−0.07	−0.41	−0.35	−0.57	0.20	−0.59	−0.35	−0.49
	*p*-value	0.001	0.001	0.001	0.001	0.001	0.001	0.001	0.001
	Sk Pearson	−0.43	0.02	−0.18	−0.40	−0.10	−0.79	−0.46	−0.36
	% km	42	50	47	44	48	33	44	32
Runner 7	Trend	↗	↘	↘	↘	↘	↘	↘	↘
	Kendall Tau	0.59	−0.73	−0.36	−0.46	−0.26	−0.47	−0.46	−0.59
	*p*-value	0.001	0.001	0.001	0.001	0.001	0.001	0.001	0.001
	Sk Pearson	−0.28	0.27	−0.60	−0.54	−0.51	−0.46	−0.85	−.65
	% km	45	53	41	42	43	42	39	43
Runner 8	Trend	↗	↘	↘	↘	↗	↘	↘	↘
	Kendall Tau	0.54	−0.46	−0.19	−0.57	0.40	−0.58	−0.26	−0.40
	*p*-value	0.001	0.001	0.001	0.001	0.001	0.001	0.001	0.001
	Sk Pearson	−0.34	0.57	0.11	−1.20	−1.33	−0.41	−0.36	−0.64
	% km	46	55	51	34	33	32	39	47
Runner 9	Trend	↗	↘	↗	↘	↗	↘	↗	↘
	Kendall Tau	0.79	−0.71	0.30	−0.51	0.77	−0.21	0.13	−0.39
	*p*-value	0.001	0.001	0.001	0.001	0.001	0.001	0.001	0.001
	Sk Pearson	0.30	−0.62	−0.11	−0.36	−0.33	−0.80	−0.08	0.55
	% km	55	42	48	42	44	40	44	53

**Table 3:** Trend characteristics of speed and cardiorespiratory variables in the marathon race (increasing: ↗, constant: ⟶ or decreasing: ↘) indexed by speed (v).

n° Runner		$\frac{\mathrm{Rf}}{v}$	$\frac{\mathrm{Vt}}{v}$	$\frac{\dot{V}E}{v}$	$\frac{\mathrm{Cadence}}{v}$	$\frac{\mathrm{HR}}{v}$	$\frac{\dot{V}O2}{v}$	$\frac{\dot{V}\mathrm{CO}2}{v}$
Runner 1	Trend	↗	↘	↘	↗	↗	↗	↘
	Kendall Tau	0.62	−0.46	−0.002	0.49	0.64	0.32	−0.22
	*p*-value	0.001	0.001	0.941	0.001	0.001	0.001	0.001
Runner 2	Trend	↗	↘	↘	↘	↗	↗	↗
	Kendall Tau	0.35	−0.58	−0.32	−0.08	0.02	−0.23	−0.41
	*p*-value	0.001	0.001	0.001	0.001	0.34	0.001	0.001
Runner 3	Trend	↗	↘	↗	↗	↗	↗	↗
	Kendall Tau	0.74	−0.50	0.53	0.26	0.64	0.44	0.19
	*p*-value	0.001	0.001	0.001	0.001	0.001	0.001	0.001
Runner 4	Trend	↗	↘	↗	↗	↗	↗	↘
	Kendall Tau	0.34	−0.30	0.07	0.26	0.38	0.02	−0.02
	*p*-value	0.001	0.001	0.001	0.001	0.001	0.001	0.302
Runner 5	Trend	↗	↗	↗	↗	↗	↗	↗
	Kendall Tau	0.55	0.07	0.26	0.54	0.53	0.37	0.08
	*p*-value	0.001	0.001	0.001	0.001	0.001	0.001	0.001
Runner 6	Trend	↗	↗	↗	↗	↗	↗	↘
	Kendall Tau	0.50	0.30	0.21	0.52	0.60	0.30	−0.04
	*p*-value	0.001	0.001	0.001	0.001	0.001	0.001	0.001
Runner 7	Trend	↗	↘	↗	↗	↗	↘	↘
	Kendall Tau	0.74	−0.48	0.06	0.45	0.57	−0.02	−0.38
	*p*-value	0.001	0.001	0.001	0.001	0.001	0.314	0.001
Runner 8	Trend	↗	↘	↗	↗	↗	↗	↘
	Kendall Tau	0.67	−0.23	0.09	0.52	0.62	0.12	−0.15
	*p*-value	0.001	0.001	0.001	0.001	0.001	0.001	0.001
Runner 9	Trend	↗	↘	↗	↗	↗	↗	↘
	Kendall Tau	0.73	−0.52	0.38	0.07	0.54	0.28	−0.13
	*p*-value	0.001	0.001	0.001	0.001	0.001	0.001	0.001

**Table 4:** Trend characteristics of speed and cardiorespiratory variables in the marathon race (increasing: ↗, constant: ⟶ or decreasing: ↘) indexed by RPE. Cad (Cadence).

n° Runner		$\frac{\mathrm{Rf}}{\mathrm{RPE}}$	$\frac{\mathrm{Vt}}{\mathrm{RPE}}$	$\frac{\dot{V}E}{\mathrm{RPE}}$	$\frac{\mathrm{Cad}}{\mathrm{RPE}}$	$\frac{\mathrm{HR}}{\mathrm{RPE}}$	$\frac{v}{\mathrm{RPE}}$	$\frac{\dot{V}O2}{\mathrm{RPE}}$	$\frac{\dot{V}\mathrm{CO}2}{\mathrm{RPE}}$
Runner 1	Trend	↘	↘	↘	↘	↘	↘	↘	↘
	Kendall Tau	−0.62	−0.84	−0.78	−0.79	−0.80	−0.79	−0.78	−0.51
	*p*-value	0.001	0.001	0.001	0.001	0.001	0.001	0.001	0.001
Runner 2	Trend	↘	↘	↘	↘	↘	↘	↘	↘
	Kendall Tau	−0.48	−0.78	−0.70	−0.74	−0.70	−0.73	−0.71	−0.74
	*p*-value	0.001	0.001	0.001	0.001	0.001	0.001	0.001	0.001
Runner 3	Trend	↘	↘	↘	↘	↘	↘	↘	↘
	Kendall Tau	−0.24	−0.83	−0.66	−0.83	−0.74	−0.78	−0.72	−0.73
	*p*-value	0.001	0.001	0.001	0.001	0.001	0.001	0.001	0.001
Runner 4	Trend	↘	↘	↘	↘	↘	↘	↘	↘
	Kendall Tau	−0.28	−0.72	−0.55	−0.82	−0.63	−0.73	−0.50	−0.54
	*p*-value	0.001	0.001	0.001	0.001	0.001	0.001	0.001	0.001
Runner 5	Trend	↘	↗	↘	↘	↘	↘	↘	↘
	Kendall Tau	−0.24	0.56	−0.38	−0.54	−0.53	−0.65	−0.43	−0.53
	*p*-value	0.001	0.001	0.001	0.001	0.001	0.001	0.001	0.001
Runner 6	Trend	↘	↘	↘	↘	↘	↘	↘	↘
	Kendall Tau	−0.29	−0.59	−0.50	−0.65	0.08	−0.64	−0.51	−0.61
	*p*-value	0.001	0.001	0.001	0.001	0.001	0.001	0.001	0.001
Runner 7	Trend	↘	↘	↘	↘	↘	↘	↘	↘
	Kendall Tau	−0.06	−0.81	−0.59	−0.62	−0.51	−0.56	−0.66	−0.70
	*p*-value	0.001	0.001	0.001	0.001	0.001	0.001	0.001	0.001
Runner 8	Trend	↘	↘	↘	↘	↘	↘	↘	↘
	Kendall Tau	−0.38	−0.68	−0.54	−0.86	−0.78	−0.81	−0.65	−0.71
	*p*-value	0.001	0.001	0.001	0.001	0.001	0.001	0.001	0.001
Runner 9	Trend	↘	↘	↘	↘	↘	↘	↘	↘
	Kendall Tau	−0.02	−0.81	−0.51	−0.82	−0.53	−0.66	−0.53	−0.70
	*p*-value	0.301	0.001	0.001	0.001	0.001	0.001	0.001	0.001

**Table 5:** Comparison of each 5-km splits (*t*-student test for paired data, mean and SD). *p* value < 0.05 (*); <0.01 (**); <0.001 (***).

		5 km	10 km	15 km	20 km	25 km	30 km	35 km	40 km	42 km
RPE	Mean	11.6	12.33	12.56	13.33	14.13	15	15.28	15.78	16.11
	SD	1.65	1.2	1.3	1.01	1.11	1.11	1.0	1.2	1.53
	Test-*t*	2.30 *	2.30 *	2.30	2.30	2.30 **	2.30 **	2.30 *	2.30 **	2.30
Cad	Mean	87.6	88.0	87.7	87.5	87.5	86.9	86.9	85.7	84.6
	SD	3.2	2.5	2.9	3.0	3.0	3.0	3.3	4.8	1.9
	Test-*t*	2.30	2.30	2.30 **	2.30	2.30	2.30 *	2.30	2.30	2.30
$\dot{V}$CO2	Mean	39.9	41.9	40.1	39.8	39.44	38.7	36.9	34.5	32.3
	SD	4.7	3.5	3.6	4.0	4.3	4.4	5.6	6.9	5.7
	Test-*t*	2.30	2.30	2.30 ***	2.30	2.30	2.30	2.30 *	2.30	2.30
$\dot{V}$O2	Mean	40.1	43.9	43.1	43.1	42.6	42.7	41.8	39.8	37.8
	SD	4.0	4.1	4.4	4.5	4.6	4.7	5.7	6.8	5.9
	Test-*t*	2.30	2.30 ***	2.30 **	2.30	2.30	2.30	2.30	2.30	2.30
Speed	Mean	12.7	13.3	13	12.9	12.7	12.3	11.9	11.18	11.43
	SD	1.0	1.1	1.2	1.2	1.2	1.3	1.4	1.6	1.8
	Test-*t*	2.30 **	2.30 ***	2.30 **	2.30	2.30	2.30	2.30 **	2.30 **	2.30
HR	Mean	150	159	159	160	162	161	160	158	160
	SD	8.7	6.18	5.35	6.84	6.16	6.23	8.05	8.4	6.9
	Test-*t*	2.30 ***	2.30 **	2.30	2.30	2.30	2.30	2.30	2.30	2.30*
RER	Mean	0.96	0.95	0.93	0.92	0.92	0.89	0.87	0.86	0.86
	SD	0.01	0.04	0.04	0.03	0.03	0.02	0.03	0.03	0.02
	Test-*t*	2.11 ***	2.30 ***	2.31 ***	2.31 ***	2.30 ***	2.30 ***	2.31 ***	2.30 ***	2.30 ***

**Table 6:** Coefficient of correlation between axis (F1 and F2) of the Principal Component Analysis and the physiological and cadence variables of the marathon time series matrix. The variance inflation factor (VIF) quantifies the severity of multicollinearity in an ordinary least squares regression analysis (see methods section).

		*Rf*	*Speed*	*Vt*	*RPE*	*HR*	Cadence	F1/F2(%)
Runner 1	F1	0.715	−0.696	−0.817	0.926	0.507	−0.242	79.6
	F2	0.566	0.617	0.328	0.085	0.768	0.729	
	VIF	3.58	3.82	4.96	3.43	1.63	5.37	
Runner 2	F1	−0.823	0.742	0.924	−0.89	0.468	0.015	75.1
	F2	0.391	0.448	−0.08	0.237	0.567	0.82	
	VIF	3.08	2.12	3.89	3.81	1.33	1.23	
Runner 3	F1	0.937	−0.372	0.826	0.943	0.764	−0.160	82.8
	F2	0.200	0.842	0.246	0.09	0.491	0.84	
	VIF	7.14	2.60	4.11	6.03	6.44	1.75	
Runner 4	F1	−0.55	0.83	0.686	−0.73	0.04	0.66	68.9
	F2	0.947	−0.78	−0.820	0.895	0.754	−0.388	
	VIF	2.23	2.96	1.68	1.93	1.76	2.43	
Runner 5	F1	−0.44	0.861	0.65	−0.801	0.449	0.44	60.2
	F2	0.76	0.21	−0.42	−0.235	0.587	−0.03	
	VIF	1.39	0.611	0.29	0.558	0.171	0.086	
Runner 6	F1	0.05	0.89	0.29	−0.72	0.706	0.906	65.7
	F2	−0.71	0.06	0.64	−0.27	−0.44	−0.09	
	VIF	1.05	3.37	1.13	1.56	1.76	3.69	
Runner 7	F1	0.61	−0.839	−0.86	0.93	−0.35	−0.82	83.3
	F2	0.74	0.32	−0.26	0.08	0.84	0.23	
	VIF	4.45	2.97	4.16	4.59	2.35	2.52	
Runner 8	F1	0.801	−0.805	−0.706	0.900	0.538	−0.731	76.0
	F2	0.439	0.359	0.04	−0.048	0.736	0.526	
	VIF	2.90	2.81	2.05	3.28	2.06	2.39	
Runner 9	F1	−0.875	0.832	0.826	0.895	−0.714	0.461	78.6
	F2	0.19	0.35	0.045	0.184	0.410	0.865	
	VIF	8.90	2.72	4.59	4.59	4.55	1.63	

## Data Availability

The data presented in this study are available on request from the corresponding author. The data are not publicly available due to privacy.

## References

[1] Run Repeat. https://runrepeat.com/state-of-running.

[2] Latta S. (2003). Hitting the wall: If you understand the scientific reasons behind “the wall”, you should be able to avoid it. Marathon Beyond.

[3] Berndsen J., Lawlor A., Smyth B. (2020). Exploring the Wall in Marathon Running. J. Sport. Anal..

[4] Smyth B. (2021). How Recreational Marathon Runners Hit the Wall: A Large-Scale Data Analysis of Late-Race Pacing Collapse in the Marathon. PLoS ONE.

[5] Smyth B. (2018). Fast Starters and Slow Finishers: A Large-Scale Data Analysis of Pacing at the Beginning and End of the Marathon for Recreational Runners. J. Sport. Anal..

[6] Billat V.L., Palacin F., Correa M., Pycke J.R. (2020). Pacing Strategy Affects the Sub-Elite Marathoner’s Cardiac Drift and Performance. Front. Psychol..

[7] Billat V.L., Petot H., Landrain M., Meilland R., Koralsztein J.P., Mille-Hamard L. (2012). Cardiac Output and Performance during a Marathon Race in Middle-Aged Recreational Runners. Sci. World J..

[8] Maron M., Horvath S.M., Wilkerson J.E., Gliner J.A. (1976). Oxygen Uptake Measurements During Competitive Marathon Running. J. Appl. Physiol..

[9] Maron M.B., Horvath S.M. (1978). The Marathon: A History and Review of the Literature. Med. Sci. Sport..

[10] Costill D.L. (1972). Physiology of marathon running. JAMA.

[11] Sjödin B., Svedenhag J. (1985). Applied Physiology of Marathon Running. Sport. Med..

[12] Coyle E.F. (2007). Physiological Regulation of Marathon Performance. Sport. Med..

[13] Joyner M.J., Hunter S.K., Lucia A., Jones A.M. (2020). Physiology and Fast Marathons. J. Appl. Physiol..

[14] Jones A.M., Kirby B.S., Clark I., Rice H.M., Fulkerson E., Wylie L.J., Wilkerson D.P., Vanhatalo A., Wilkins B.W. (2021). Physiological Demands of Running at 2-h Marathon Race Pace. J. Appl. Physiol..

[15] Utter A.C., Kang J., Robertson R.J., Nieman D.C., Chaloupka E.C., Suminski R.R., Piccinni C.R. (2002). Effect of Carbohydrate Ingestion on Ratings of Perceived Exertion During a Marathon. Med. Sci. Sport. Exerc..

[16] Borg G. (1998). Borg’s Perceived Exertion and Pain Scales.

[17] Mauger A.R. (2014). Factors Affecting the Regulation of Pacing: Current Perspective. J. Sport. Med..

[18] Pycke J.R., Billat V. (2022). Marathon Performance Depends on Pacing Oscillations between Non Symmetric Extreme Values. Int. J. Environ. Res. Public Health.

[19] Kendall M.G., Stuart A. (1963). The Advance Theory of Statistics.

[20] Billat V., Carbillet T., Correa M., Pycke J.-R. (2019). Detecting the Marathon Asymmetry with a Statistical Signature. Phys. A Stat. Mech. Appl..

[21] Billat V., Vitiello D., Palacin F., Correa M., Pycke J.R. (2020). Race Analysis of the World’s Best Female and Male Marathon Runners. Int. J. Environ. Res. Public Health.

[22] Schroeder M.A. (1990). Diagnosing and dealing with multicollinearity. West. J. Nurs. Res..

[23] Muñoz-Pérez I., Mecías-Calvo M., Crespo-Álvarez J., Sámano-Celorio M.L., Agudo-Toyos P., Lago-Fuentes C. (2020). Different Race Pacing Strategies Among Runners Covering the 2017 Berlin Marathon under 3 h and 30 min. PLoS ONE.

[24] Breen D., Norris M., Healy R., Anderson R. (2018). Marathon Pace Control in Masters Athletes. Int. J. Sport. Physiol. Perform..

[25] Nikolaidis P.T., Knechtle B. (2018). Pacing Strategies in the ’Athens Classic Marathon’: Physiological and Psychological Aspects. Front. Physiol..

[26] Myrkos A., Smilios I., Kokkinou E.M., Rousopoulos E., Douda H. (2020). Physiological and Race Pace Characteristics of Medium and Low-Level Athens Marathon Runners. Sport.

[27] Morgan W.P. (1994). Psychological Components of Effort Sense. Med. Sci. Sport. Exerc..

[28] Foster C., Hendrickson K.J., Peyer K., Reiner B., Dekoning J.J., Lucia A., Battista R.A., Hettinga F.J., Porcari J.P., Wright G. (2009). Pattern of developing the performance template. Br. J. Sport. Med..

[29] Noakes T.D. (2008). Rating of Perceived Exertion as a Predictor of the Duration of Exercise that Remains Until Exhaustion. Br. J. Sport. Med..

[30] Lambert E.V., St Clair Gibson A., Noakes T.D. (2005). Complex Systems Model of Fatigue: Integrative Homoeostatic Control of Peripheral Physiological Systems During Exercise in Humans. Br. J. Sport. Med..

[31] Ranum M., Foster C., Cami C., Wright G., Guidotti F., De Koning J.J., Dodge C., Porcari J.P. (2021). Effect of Running Velocity Variation on the Aerobic Cost of Running. Int. J. Environ. Res. Public Health.

[32] Crandall C.G., González-Alonso J. (2010). Cardiovascular Function in the Heat-Stressed Human. Acta Physiol..

[33] Fritzsche R.G., Switzer T.W., Hodgkinson B.J., Coyle E.F. (1999). Stroke Volume Decline During Prolonged Exercise Is Influenced by the Increase in Heart Rate. J. Appl. Physiol..

[34] Billat V.L., Dupre M., Karp J.R., Koralsztein J.P. (2010). Mountaineering Experience Decreases the Net Oxygen Cost of Climbing Mont Blanc (4808 m). Eur. J. Appl. Physiol..

[35] Meyer F., Falbriard M., Mariani B., Aminian K., Millet G.P. (2021). Continuous Analysis of Marathon Running Using Inertial Sensors: Hitting Two Walls?. Int. J. Sport. Med..

[36] Doherty C., Keogh A., Davenport J., Lawlor A., Smyth B., Caulfield B. (2020). An Evaluation of the Training Determinants of Marathon Performance: A Meta-Analysis with Meta-Regression. J. Sci. Med. Sport..

